# *Akkermansia muciniphila* Exerts Strain-Specific Effects on DSS-Induced Ulcerative Colitis in Mice

**DOI:** 10.3389/fcimb.2021.698914

**Published:** 2021-08-04

**Authors:** Qing Liu, Wenwei Lu, Fengwei Tian, Jianxin Zhao, Hao Zhang, Kan Hong, Leilei Yu

**Affiliations:** ^1^State Key Laboratory of Food Science and Technology, Jiangnan University, Wuxi, China; ^2^School of Food Science and Technology, Jiangnan University, Wuxi, China; ^3^National Engineering Research Center for Functional Food, Jiangnan University, Wuxi, China; ^4^Wuxi Translational Medicine Research Center and Jiangsu Translational Medicine Research Institute Wuxi Branch, Wuxi, China; ^5^Wuxi People’s Hospital Affiliated to Nanjing Medical University, Wuxi, China

**Keywords:** *Akkermansia muciniphila*, ulcerative colitis, intestinal inflammation, comparative genomics, strain-specific

## Abstract

*Akkermansia muciniphila* is a commensal bacterium of the gut mucus layer. Although both *in vitro* and *in vivo* data have shown that *A. muciniphila* strains exhibit strain-specific modulation of gut functions, its ability to moderate immunity to ulcerative colitis have not been verified. We selected three isolated human *A. muciniphila* strains (FSDLZ39M14, FSDLZ36M5 and FSDLZ20M4) and the *A. muciniphila* type strain ATCC BAA-835 to examine the effects of different *A. muciniphila* strains on dextran sulfate sodium-induced colitis. All of the *A. muciniphila* strains were cultured anaerobically in brain heart infusion medium supplemented with 0.25% type II mucin from porcine stomach. To create animal models, colitis was established in C57BL/6 mice which randomly divided into six groups with 10 mice in each group by adding 3% dextran sulfate sodium to drinking water for 7 days. *A. muciniphila* strains were orally administered to the mice at a dose of 1 × 10^9^ CFU. Only *A. muciniphila* FSDLZ36M5 exerted significant protection against ulcerative colitis (UC) by increasing the colon length, restoring body weight, decreasing gut permeability and promoting anti-inflammatory cytokine expression. However, the other strains (FSDLZ39M14, ATCC BAA-835 and FSDLZ20M4) failed to provide these effects. Notably, *A. muciniphila* FSDLZ20M4 showed a tendency to exacerbate inflammation according to several indicators. Gut microbiota sequencing showed that *A. muciniphila* FSDLZ36M5 supplementation recovered the gut microbiota of mice to a similar state to that of the control group. A comparative genomic analysis demonstrated that the positive effects of *A. muciniphila* FSDLZ36M5 compared with the FSDLZ20M4 strain may be associated with specific functional genes that are involved in immune defense mechanisms and protein synthesis. Our results verify the efficacy of *A. muciniphila* in improving UC and provide gene targets for the efficient and rapid screening of *A. muciniphila* strains with UC-alleviating effects.

**Figure:**
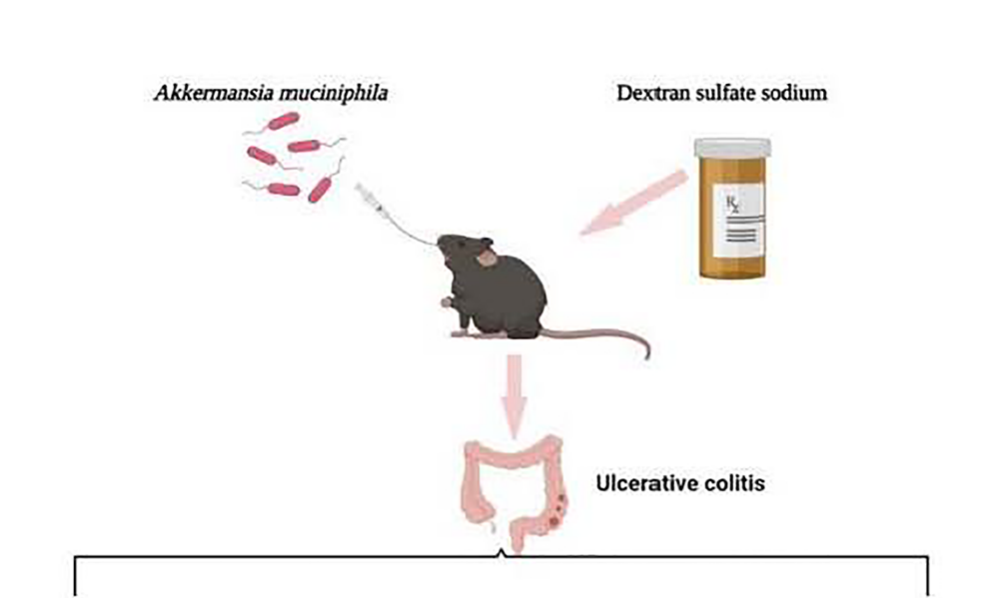
Graphical Abstract

## Introduction

*Akkermansia muciniphila* is a strictly anaerobic gram-negative gut bacterium that uses mucin as its sole energy source (Derrien et al., 2017). This bacterium is a potential candidate for next-generation probiotic because it has a positive effect on human metabolic disorders such as diabetes, alcoholic liver disease, obesity and gastrointestinal diseases (Cani and de Vos, 2017; Plovier et al., 2017; Grander et al., 2018; Bian et al., 2019).

Increasing numbers of studies are focusing on the relationship between *A. muciniphila* and intestinal barrier function and host immunity. For example, Png et al. demonstrated that the abundance of *A. muciniphila* was dramatically decreased in patients with inflammatory bowel disease compared with controls, suggesting that *A. muciniphila* may be correlated with intestinal mucosal health (Png et al., 2010). Several reports have observed that *A. muciniphila* is associated with body weight gain, reduction in the expression of pro-inflammatory cytokines and the recovery of gut epithelial barrier function in dextran sulfate sodium (DSS)-induced colitis (Bian et al., 2019; Zhai et al., 2019). Other studies have shown that supplementation with *A. muciniphila* can restore the normal thickness of the inner mucus layer and increase the expression of tight-junction (TJ) proteins such as claudins, occludin and zonula occludens-1(ZO-1) in the gut of mice (Everard et al., 2013; Geerlings et al., 2018; Grander et al., 2018). These findings suggest that *A. muciniphila* plays an important role in protecting the intestinal tract from damage.

Although multiple reports have suggested that probiotics have the potential to alleviate intestinal disease, some studies have demonstrated that the effects of probiotics on intestinal disease are strain specific. For example, *Lactobacillus casei* ATCC BAA-835 393, DN-114001 and Lbs2 have been demonstrated to alleviate damage in gut disease by restoring histopathological damage, improving the disease activity index (DAI) score, increasing the differentiation of regulatory T (Treg) cells and inhibiting pro-inflammatory cytokine expression (Zakostelska et al., 2011; Thakur et al., 2016; Xu et al., 2018). However, other reports have revealed that the ingestion of *L. casei* does not prevent the occurrence of diarrhea or inhibit pathogenic infections (Arena et al., 2017). Moreover, a study found that *in vitro A. muciniphila* ATCC BAA-835 significantly augmented Treg differentiation, whereas another *A. muciniphila* strain 139 did not have this effect (Zhai et al., 2019). Another study showed that some murine *A. muciniphila* strains may aggravate colitis in IL10−/− mice (Seregin et al., 2017). In sum, these findings suggest that different *A. muciniphila* strains have strain-specific physiological functions.

Studies have shown that confounding factors including the origin, genetic background and physiological characteristics of the strains may influence the functional specificity of probiotics. Different species are able to adjust to specific environments through genome specialization that drive niche‐specific adaptations (Frese et al., 2012; Cen et al., 2020; Xiao, 2020). Specific genes in the gut bacteria determine their beneficial function in the intestine. One study conducted a phylogenetic genomic analysis of 100 strains of *L. rhamnosu*s isolated from different sources and found that the differences among the strains were partly related to the differing ecological niches they occupied, resulting in strains with different competitive advantages in different environments that thus exert different probiotic effects (Frese et al., 2012). Furthermore, the intestinal protective effects of probiotic bacterial strains are closely related to their physiological characteristics. A study has claimed that the micro integral membrane proteins of *L. plantarum* CGMCC 1258 can remedy TJ injury by upregulating the relative expression of TJ proteins such as JAM-1and claudin-1 (Yin et al., 2018). Amuc_1100, a membrane protein secreted by *A. muciniphila*, can remedy metabolic disorders and enhance the intestinal permeability of mice (Plovier et al., 2017). Extracellular vesicles of *A. muciniphila* ATCC BAA-835 have been demonstrated to have a positive effect on alleviating DSS-induced colitis in mice (Kang et al., 2013). Furthermore, conjugated linoleic acids and short-chain fatty acids produced by probiotics also improve the gut barrier and regulate gut immune function (Cremon et al., 2018).

Although much evidence has shown that probiotics have a beneficial effect on colitis, the positive effects of *A. muciniphila* and its ability to moderate immunity to colitis have not been verified. The inter-species or inter-genetic differences of *A. muciniphila* has been evaluated by several studies, but no definite conclusion has been reached thus far, partly due to the limited number of isolated strains of this species. In fact, most investigations of *A. muciniphila* have only assessed the type strain ATCC BAA-835. Therefore, we selected *A. muciniphila* ATCC BAA-835 and three other *A. muciniphila* strains, FSDLZ36M5, FSDLZ39M14 and FSDLZ20M4, isolated from human feces in our laboratory to investigate the effects of *A. muciniphila* on DSS-induced ulcerative colitis (UC). The genomic and functional characteristics of the different *A. muciniphila* strains were used to assess their effects from a genetic perspective.

## Materials and Methods

### Bacterial Strains and Preparation

All of the *A. muciniphila* strains, namely ATCC BAA-835, FSDLZ20M4, FSDLZ36M5 and FSDLZ39M14, were cultured anaerobically (10% H_2_, 10% CO_2_ and 80% N_2_) in synthetic media, the machine for anaerobic culture incubator (GRP-9080) was purchased from Shanghai senxin Experimental Instrument Company (Shanghai, China). The strain was cultured in brain heart infusion (BHI) medium (Qingdao Hope Bio-Tcehnology Company, Qingdao, China) supplemented with 0.25% type II mucin from porcine stomach (Sigma) at 37°C for 18 h.

### Animal Experiment Design

Sixty adult male specific pathogen-free C57BL/6 mice were randomly divided into six groups with 10 mice in each group: five of the groups were administered DSS to induce UC (UC groups) and the sixth was a control group. Four of the UC groups were supplemented with one of the strains of *A. muciniphila* (treatment groups) and the fifth received no supplementation (DSS group). All of the mice were fed standard commercial mouse food and kept in a 12-h light/dark cycle environment at 22–24°C under controlled humidity (40–70%). The experimental protocol was approved by the Ethics Committee of Jiangnan University, China (JN. No.20190915c0801101), and was performed in accordance with European Community Directive 2010/63/EU.

Each mouse in the control group was administered 0.2 ml of sterile phosphate-buffered saline (PBS) daily and distilled water during the 7-day experiment. The other five groups were given 3% (w/v) DSS (36–50 kDa, MP Biomedicals Ltd, Santa Ana, USA) in drinking water to induce UC, and those in the treatment groups were given a sterile PBS suspension of *A. muciniphila* (1 × 10^9^ CFU/0.2 ml per mouse per day). After 7 days of treatment, the mice were anesthetized and sacrificed. The feces, blood and colon tissues of the mice were then harvested for analysis. The six groups were as follows.

Control group: Sterile PBS + distilled water
DSS group: DSS + sterile PBS
ATCC BAA-835: DSS + *A. muciniphila* ATCC BAA-835 bacterial suspension
FSDLZ20M4: DSS + *A. muciniphila* FSDLZ20M4 bacterial suspension
FSDLZ36M5: DSS + *A. muciniphila* FSDLZ36M5 bacterial suspension
FSDLZ39M14: DSS + *A. muciniphila* FSDLZ39M14 bacterial suspension

### Assessment of the Severity of UC

During the experiment, the body weight of each mouse was measured daily and the colon length was measured when the mice were sacrificed to determine the severity of the induced UC (Yang et al., 2020).

### Gut Permeability

The fluorescein isothiocyanate-conjugated dextran (FITC-dextran) assay obtained from Sigma-Aldrich (Saint Louis, MO, USA) was used to determine the gut permeability of the mice. The concentration of FITC-dextran was determined using a previously described method (Bian et al., 2019).

### Biochemical Analysis of the Colon Tissue

A colon sample from each mouse was accurately weighed (0.1 g) and homogenized in nine volumes of cold PBS. The supernatant was separated by centrifugation at 3000 g (4°C, 5 min). The mucin 2 (MUC2), interleukin (IL)-6, IL-10, IL-1β and tumor necrosis factor alpha (TNF-α) contents were detected using the corresponding kits from SenBeiJia Biological Technology Ltd. (Nanjing, China).

### Fecal DNA Extraction, Sequencing, and Analysis

The bacterial DNA from mouse feces was isolated using a FastDNA^®^ Spin kit from MP Biomedicals Ltd. (CA, USA). The sequencing of the intestinal microbiota was performed using a previously described method. A principal component analysis (PCA) was used with the STAMP software to analyze the results. The inter-group differences in the intestinal microbiota composition were determined by an LEfSe analysis.

### Comparative Genomic Analysis of *A. muciniphila*

The genome sequencing and annotation of the *A. muciniphila* isolates were performed following the method given in previous studies. Maximum likelihood phylogenetic tree using the neighbor-joining method was analyzed and constructed using FastTree (Price et al., 2010). Orthologous genes were generated using Roary with the default parameters (Page et al., 2015). COG annotation was done with the COG database using BLAST (Tatusov et al., 2000). The CAZyme database (Lombard et al., 2014) were used to annotate carbohydrate active enzymes.

### Statistical Analysis

Experimental data are presented as the mean ± standard error of the mean. A one-way analysis of variance (ANOVA) was used to analyze the data, followed by Tukey’s multiple comparison test to identify statistical significance. *P* values < 0.05 were regarded as statistically significant. All of the statistical analyses were conducted and visualized with GraphPad Prism (GraphPad Software Inc., San Diego, CA, USA).

## Results

### Phylogenetic Tree of the *A. muciniphila* Strains

**Figure 1:**
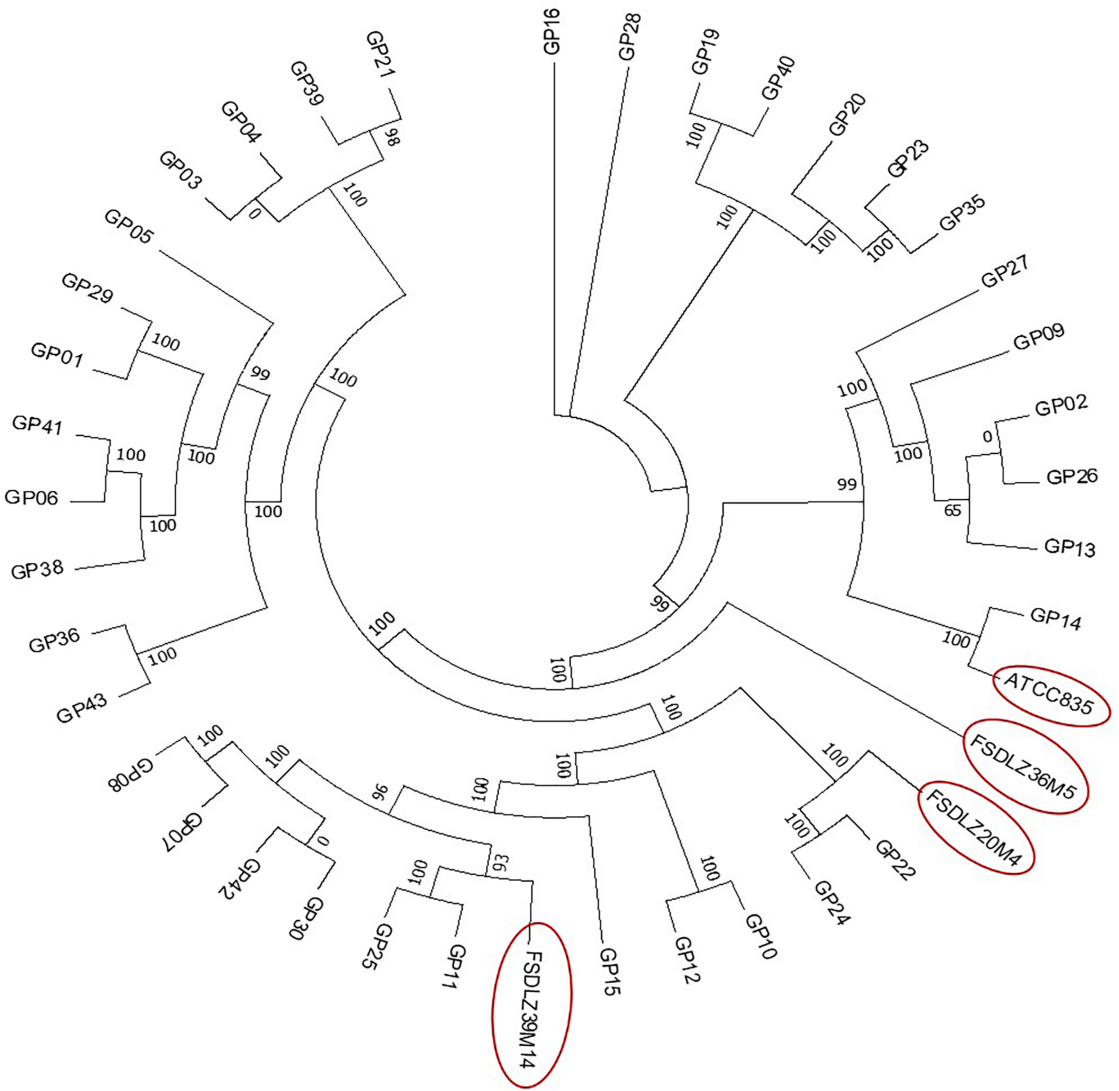
Maximum likelihood phylogenetic tree based on the core genomes of 40 *A. muciniphila* isolates. The neighbor-joining method was used to infer the evolutionary history of the core-genomes of the 40 *A. muciniphila* strains.

We selected 37 reported *A. muciniphila* strains (including the type strain ATCC BAA-835) and 3 *A. muciniphila* strains from our laboratory to analyze the evolutionary history of *A. muciniphila*, all of which were isolated from fecal samples from Chinese people. The 40 strains shared 598 homologous genes. We found that FSDLZ36M5, FSDLZ20M4 and FSDLZ39M14 occurred on different branches of the phylogenetic tree (**Figure 1**). Therefore, we selected the type strain and these three strains with relatively distant genetic relationships to evaluate their ability to alleviate UC.

### Effect of *A. muciniphila* on Body Weight and Colon Length

**Figure 2:**
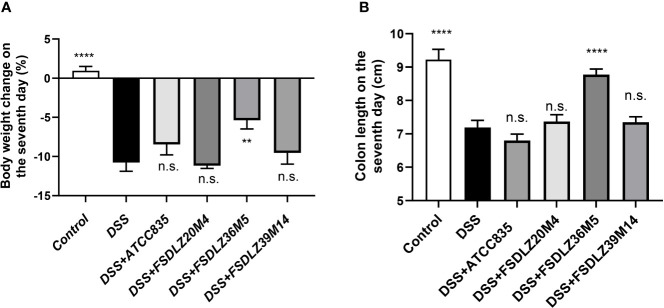
Effects of *A. muciniphila* strains on a DSS-induced ulcerative colitis mouse model. **(A)** Body weight change after 7 days of DSS intake (%). **(B)** Colon length after 7 days of DSS intake. **P < 0.01 *vs* the DSS group, ****P < 0.0001 *vs* the DSS group. DSS, dextran sulfate sodium. n.s., not significant.

The addition of DSS to the drinking water of the mice in the UC groups resulted in a significant drop in body weight and a rapid shortening of the colon relative to that of the control group (**Figures 2A, B**). After supplementation with *A. muciniphila* FSDLZ36M5, all indicators showed significant improvement compared with those of the untreated DSS group. However, the other strains did not show any obvious alleviating effects, and *A. muciniphila* FSDLZ20M4 had a tendency to exacerbate the physiological damage to the gut of the mice.

### Effect of *A. muciniphila* on Gut Permeability

**Figure 3:**
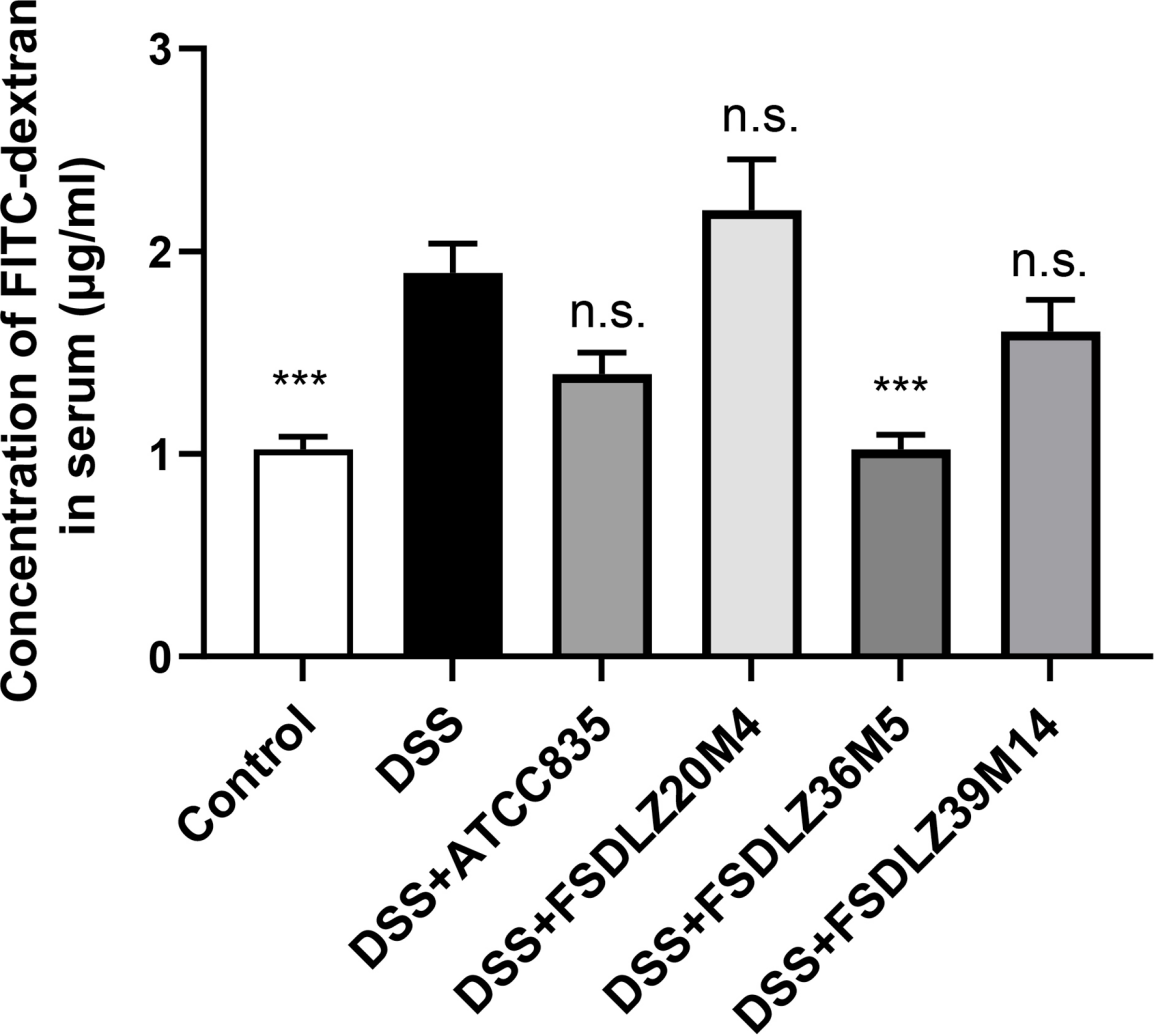
Gut permeability determined by measuring the plasma concentration of FITC-dextran. ***P < 0.001 *vs* the DSS group. FITC-dextran, fluorescein isothiocyanate conjugated dextran; DSS, dextran sulfate sodium. n.s., not significant.

DSS caused significant increases in the concentration of FITC-dextran in the serum of the mice in the UC groups compared with the control group (*P* < 0.001, **Figure 3**). Treatment with different *A. muciniphila* had different effects on the increased gut permeability caused by DSS. A reduced gut permeability in mice treated with *A. muciniphila* ATCC BAA-835, FSDLZ36M5 and FSDLZ39M14 was observed relative to the untreated DSS group, and *A. muciniphila* FSDLZ36M5 in particular contributed to a marked reduction in the levels of FITC-dextran (*P* < 0.001, **Figure 3**), indicating enhanced gut barrier function. However, the oral administration of *A. muciniphila* FSDLZ20M4 tended to aggravate the damage to the intestinal barrier of the mice.

### Effect of *A. muciniphila* on Inflammatory Cytokine Expression in the Colon

**Figure 4:**
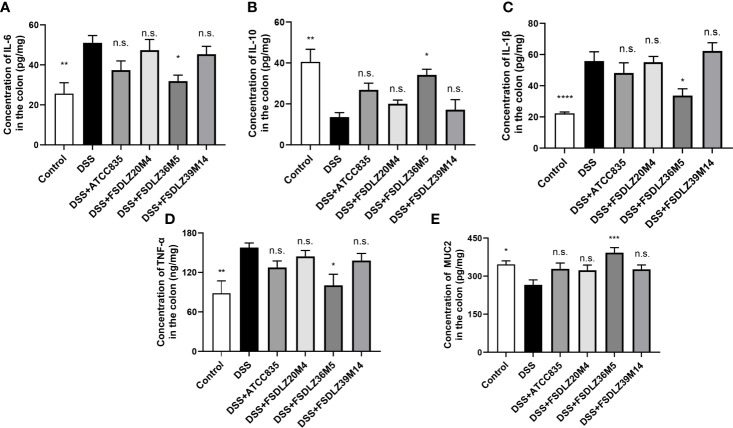
Effects of *A. muciniphila* on the expression of inflammatory cytokines and MUC2 in colon tissue. Bar charts represent the colonic cytokine levels of IL-6 **(A)**, IL-10 **(B)**, IL-1β **(C)**, TNF-α **(D)** and MUC2 **(E)** in the groups. *P < 0.05 *vs* the DSS group, **P < 0.01 *vs* the DSS group, ****P < 0.0001 *vs* the DSS group. DSS, dextran sulfate sodium; IL, interleukin; TNF-α, tumor necrosis factor alpha; MUC2, mucin 2. n.s., not significant. ***P < 0.001 vs the DSS group.

DSS led to the substantial expression of inflammatory cytokines in the colon tissue (**Figure 4**). An increase in pro-inflammatory cytokines (TNF-α, IL-1β and IL-6) and a decrease in anti-inflammatory cytokines (IL-10) were observed in all of the mice administered with DSS compared with the control group (**Figures 4A–D**). After treatment with *A. muciniphila* FSDLZ36M5, all cytokine indicators significantly improved (*P* < 0.05). Similar results were found for the three other *A. muciniphila* strains (**Figure 4B**). Furthermore, compared with the control group, the MUC2 content in the DSS group was significantly decreased, but the content was recovered in the treated groups, consistent with the above (**Figure 4E**).

### Effect of *A. muciniphila* on the Gut Microbiota Composition

**Figure 5:**
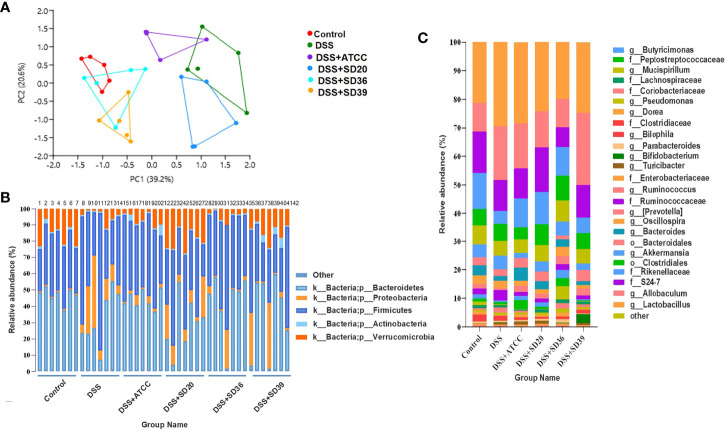
The relative abundance analysis of microbiome composition in ulcerative colitis-induced mice. **(A)** Principal component analysis of the gut microbiota. Each plot represents one sample. **(B)** Relative abundance of taxa at the phylum level. **(C)** Relative abundance of taxa at the genus level.

**Figure 6:**
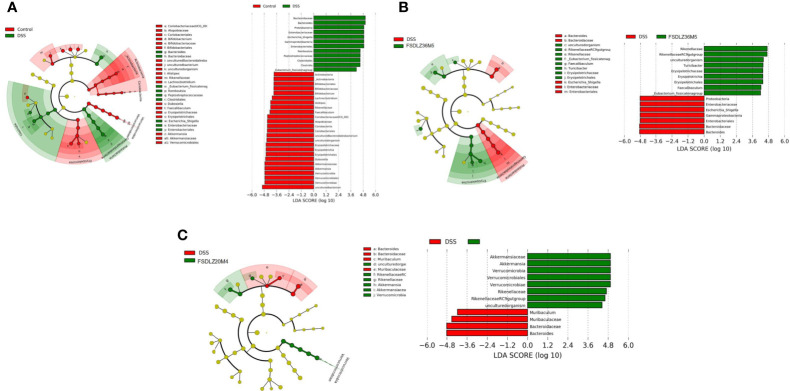
LEfSe analysis of gut microbiome composition in ulcerative colitis-induced mice. **(A)** LEfSe analysis of the DSS group. **(B)** LEfSe analysis of the *A. muciniphila* FSDLZ36M5 group. **(C)** LEfSe analysis of the *A. muciniphila* FSDLZ20M4 group. DSS, dextran sulfate sodium.

The PCA results revealed that the gut microbiota compositions of the UC groups were significantly different from that of the control group (**Figure 5A**). Supplementation with *A. muciniphila* strains led to structural alterations in the gut microbial communities of all of the treated groups relative to the gut microbiota of the untreated DSS group. Notably, the gut microbiota of the group supplemented with the *A. muciniphila* FSDLZ36M5 strain was similar to that of the control group, but the other three strains had no prominent recovery effect (**Figure 5A**). At the phylum level, in the DSS group, the abundance of Firmicutes and Proteobacteria significantly increased, whereas that of Verrucomicrobia and Actinobacteria decreased to varying degrees compared with the control group (**Figure 5B**). At the genus level, the abundance of *Bifidobacterium* and *Lactobacillus* was recovered in the *A. muciniphila* FSDLZ36M5 group (**Figure 5C**) to levels greater than those in the DSS group. Meanwhile, a significant drop in the abundance of *Bacteroides* and *Enterobacteriaceae* was observed (**Figure 5C**). The LEfSe analysis also revealed similar results (**Figures 6A–C**). 

### Comparative Genomic Analysis of the Selected *A. muciniphila* Strains

**Figure 7:**
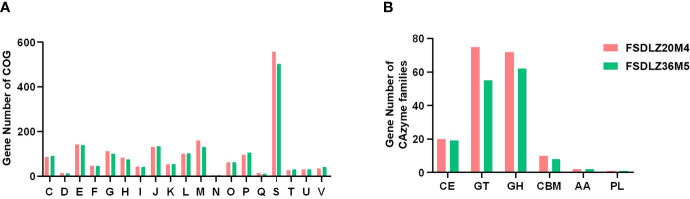
Clusters of orthologous groups (COGs) repertoires **(A)** and carbohydrate-active enzyme (CAZy) families **(B)** of *A. muciniphila* FSDLZ36M5 and *A. muciniphila* FSDLZ20M4.

Although there was no significant difference in the cluster of orthologous groups (COGs) and carbohydrate-active enzyme (CAZy) levels between FSDLZ36M5 and FSDLZ20M4 strains (**Figures 7A, B**), there were some specific genes in the FSDLZ36M5 strain (**Table 1**).

**Table 1:** Strain-specific orthologous groups (COGs) categories of *Akkermansia muciniphila* FSDLZ36M5 compared to *A. muciniphila* FSDLZ20M4.

Gene name	COG ID	Description	COG type
metE	COG0620	5-methyltetrahydropteroyltriglutamate–homocysteine methyltransferase	E
rpsL	COG0048	ribosomal protein S12	J
rpmB	COG0227	50S ribosomal protein L28	J
arsR	ENOG411226U	transcriptional regulator ArsR family	K
mod	COG2189	site-specific DNA-methyltransferase	L
cas2	COG3512	cRISPR-associated endoribonuclease Cas2	L
dcm	COG0270	DNA (cytosine-5-)-methyltransferase	L
csd1	ENOG410ZVNC	CRISPR-associated protein, Csd1 family	L
cwlK	ENOG4111NIG	peptidase M15B and M15C DD-carboxypeptidase VanY/endolysin	M
cysNC	COG2895	sulfate adenylyltransferase	P
cysD	COG0175	sulfate adenylyltransferase subunit 2	P
cheY	COG0784	response regulator	T
kdpD	COG2205	two-component sensor histidine kinase	T
res	COG3587	type III restriction endonuclease subunit R	V
cpt	COG3896	chloramphenicol phosphotransferase	V
hsdR	COG4096	Type I site-specific restriction-modification system, R (restriction) subunit and related helicases	V
secE	ENOG410Y6UA	preprotein translocase subunit SecE	U

## Discussion

This study aimed to verify whether the UC-alleviating effects of *A. muciniphila* are strain specific. Although the evidence of functional differences between different strains of this species is abundant, the factors that contribute to the specificity of these strains remain unclear. The genetic background of the bacterial strains is considered to have an important influence on the functional specificity of probiotics (Ribbera et al., 2013; Luongo et al., 2017; Wang et al., 2017). A previous report revealed that although the genomes of *A. muciniphila* ATCC BAA-835 and *A. muciniphila* 139 are highly similar, they contain strain-specific genes that result in a differing ability to relieve gut inflammation and restore intestinal flora in chronic colitis (Zhai et al., 2019). It was also reported that *L. kefiranofaciens* ZW3 showed a stronger ability to protect the gut barrier than other investigated strains of the same species due to the presence of four specific genes (*pgm*, *ugp*, *uge* and *pgi*), which were identified to encode enzymes that regulate carbon flux and in turn affect exopolysaccharides yield, thus exerting a positive effect on the host gut microbiota (Xing et al., 2017). Furthermore, a study showed that *Bifidobacterium infantis* EVC001 had a remarkable fitness advantage over other tested strains due to the presence of an H5 gene cluster associated with the ability to metabolize human milk oligosaccharides and to colonize the infant gut, resulting in the strain having a beneficial function in the gut of infants (Duar et al., 2020). We therefore selected four *A. muciniphila* strains with relatively different genetic positions on the phylogenetic tree as the test strains to evaluate their ability to alleviate UC.

Our results verified that the therapeutic effect of *A. muciniphila* strains on UC was strain specific. Among the four *A. muciniphila* strains used in the animal study, *A. muciniphila* FSDLZ36M5 showed a positive effect on UC symptoms, but the other tested strains failed to show such effects. Two indicators, body weight and colon length, were used to determine the severity of UC. Most previous studies have found increased gut permeability and a thinner colonic mucus layer in mouse and human patients with UC, indicating impairment of the gut barrier that exacerbates UC (Petersson et al., 2011; Dicksved et al., 2012). *A. muciniphila* is considered to be a mucin-degrading bacteria that has the ability to continuously refresh and reshape the gut mucus layer, thus improving the integrity of the gut epithelial barrier and maintaining intestinal homeostasis (Derrien et al., 2008; Everard et al., 2013). *In vivo*, we observed a decreased concentration of FITC-dextran in the blood and increased MUC2 content in the colon tissue of mice due to *A. muciniphila* supplementation, which indicated decreased permeability and restored gut barrier function, in accordance with the results of previous reports (Bian et al., 2019).

Furthermore, several studies have reported that UC may be linked with an overreaction of the immune system to the gut microbiota (Wang et al., 2017; Zhang et al., 2017). Hence, the gut microbial composition of each group in this study was analyzed to investigate the effect of each strain of *A. muciniphila* on the gut microbiome. Our findings revealed an altered gut microbiota in the mice treated with DSS, with a change in the relative abundance of specific bacterial taxa. After the administration of *A. muciniphila* FSDLZ36M5, we found changes in the fecal bacteria, such as increased *Lactobacillus* abundance and decreased *Enterobacteriaceae* abundance. Specific species in the *Lactobacillus* genus have been found to protect the gut from inflammatory damage and augment the gut barrier (Simeoli et al., 2015; Zhang et al., 2018; Li et al., 2020; Panpetch et al., 2020). Certain *Enterobacteriaceae* species are enriched in mice and in human patients with inflammatory bowel disease, and *Escherichia* species have also been found in ileal biopsies of patients with UC (Meng et al., 2018). Notably, augmented *Escherichia* abundance may aggravate disease severity by increasing gut permeability. We observed similar findings. In previous studies, most of the regulatory effects of *A. muciniphila* on UC were mainly found with the type strain ATCC BAA-835, with some studies showing that gavage of *A. muciniphila* strain ATCC BAA-835 effectively ameliorated the adverse effects of DSS-induced colitis (Bian et al., 2019). However, in our study, ATCC BAA-835 did not seem to have a significant therapeutic effect. This result is consistent with that of a previous study by Kang et al. (2013). We speculate that the number of strains, the phase of colitis in mice and sex of the mice may influence the effects of the *A. muciniphila* strain ATCC BAA-835. In our experiment, the bacterial dose in the treated groups was 1 × 10^9^ CFU/0.2 ml, whereas in previous studies, the dose was 1 × 10^9^ CFU/ml, which may explain the differences in the UC-alleviating effects in this study (Bian et al., 2019). A previous study also reported that different doses of probiotics had significantly different effects on reducing the colonization of *Salmonella enterica* serovar Typhimurium in the intestine, with high doses resulting in a significant decrease in *S. enterica* serovar Typhimurium counts in the intestine relative to low doses, thus protecting gut barrier function (Haghighi et al., 2008). Further, the pathological characteristics of DSS-induced acute UC and chronic UC are not the same, so the performance of bacterial strains in these two models will also be different (Perše 2012). Moreover, sex of the mouse models has been revealed to affect the pathogenicity of DSS-induced colitis (Wagnerova et al., 2017). Due to the different colonization ability of intestinal microorganisms in the gastrointestinal tract, their beneficial effects could be affected.

Finally, the potential genetic background of *A. muciniphila* isolates can be explained by comparative genomic analysis. Nineteen strain-specific COG categories were detected in the strain that showed significant protective effects against colitis (*A. muciniphila* FSDLZ36M5) but not in the strain that showed negative effects (*A. muciniphila* FSDLZ20M4; **Table 1**). Among these genes, *res*, *cpt*, and *hsdR* have been reported to be involved in immune defense mechanisms that help bacterial strains develop self-defense in harsh environments, thus giving them a colonization advantage (Kholodii et al., 1995; Ferri et al., 2010; Morash et al., 2010). A previous study found that certain adverse conditions can induce the expression of several key stress and resistance genes/proteins in bacteria, including chloramphenicol phosphotransferase encoded by *cpt*, which can help bacteria colonize and develop resistance in adverse ecological environments (Hassan et al., 2019). It has been reported that the adaptability of probiotics to environmental conditions in the gastrointestinal tract is important to their residence time and survival rate (Saulnier et al., 2011). This property is consistent with probiotic performance in DSS-induced colitis. The gene *metE* detected in *A. muciniphila* FSDLZ36M5 is responsible for methionine formation, an essential amino acid that supports protein synthesis and the regulation of the gut mucosal immune response and barrier function (Bauchart-Thevret et al., 2009). This specific gene is also present in *Lactobacillus helveticus* MTCC 5463, and can protect against oxidative stress induced by macrophages and prevent intestinal mucosal cell damage (Senan et al., 2015). In agreement with this finding, supplementation with *A. muciniphila* FSDLZ36M5 in this study significantly increased the MUC2 content and decreased the expression of inflammatory cytokines compared with the other test strains (*P* < 0.05, **Figure 4D**). Our results suggest that the integration of key phenotypic and genotypic characteristics of strains may be an effective method to screen for strains that are adapted to specific functions that could be clinically exploited. In addition, it has been reported selective colonization ability of human fecal microbes in different mouse gut environments.

## Conclusions

Overall, our study confirms that the beneficial effects of *A. muciniphila* in DSS-induced UC are strain specific. Among the four *A. muciniphila* strains used in the animal study, *A. muciniphila* FSDLZ36M5 showed a positive effect on UC symptoms, but the other tested strains failed to show such effects. Firstly, on physiological indicators including colon length and body weight, *A. muciniphila* FSDLZ36M5 showed significant improvement compared with those of the untreated DSS group. Besides, *In vivo*, we observed a decreased concentration of FITC-dextran in the blood and increased MUC2 content in the colon tissue of mice due to *A. muciniphila* FSDLZ36M5 supplementation, which indicated decreased permeability and restored gut barrier function. Moreover, after treatment with *A. muciniphila* FSDLZ36M5, all cytokine indicators (TNF-α, IL-1β and IL-6) significantly improved. Furthermore, notably, the gut microbiota of the group supplemented with the *A. muciniphila* FSDLZ36M5 strain was similar to that of the control group. We have added related contents in the *Discussion* section. In addition, comparative genome analysis detected a series of specific genes related to immune defense mechanisms and methionine transport and metabolism that have been reported to regulate the mucosal immune response and protect the intestinal epithelial barrier, and thus play a positive role in alleviating UC.

## Data Availability Statement

The datasets presented in this study can be found in online repositories. The names of the repository/repositories and accession number(s) can be found below: https://www.ncbi.nlm.nih.gov/, PRJNA714373.

## Ethics Statement

The animal study was reviewed and approved by the Ethics Committee of Jiangnan University, China (JN. No. 20190915c0801101).

## Author Contributions

QL: Methodology, Software, Formal analysis, Visualization, Writing-original draft. WL: Methodology, Software. FT: Investigation, Formal analysis. JZ: Validation, Investigation. HZ: Validation, Investigation. KH: Conceptualization, Validation, Investigation. LY: Conceptualization, Writing-Project administration, Validation, Investigation. All authors contributed to the article and approved the submitted version.

## Funding

This work was supported by the Natural Science Foundation of Jiangsu Province [BK20200084]; The National Natural Science Foundation of China [No. 32021005 and No. 31820103010]; The National first-class discipline program of Food Science and Technology [JUFSTR20180102]; and Collaborative innovation center of food safety and quality control in Jiangsu Province.

## Conflict of Interest

The authors declare that the research was conducted in the absence of any commercial or financial relationships that could be construed as a potential conflict of interest.

## Publisher’s Note

All claims expressed in this article are solely those of the authors and do not necessarily represent those of their affiliated organizations, or those of the publisher, the editors and the reviewers. Any product that may be evaluated in this article, or claim that may be made by its manufacturer, is not guaranteed or endorsed by the publisher.
